# A database of global coastal conditions

**DOI:** 10.1038/s41597-021-01081-9

**Published:** 2021-11-26

**Authors:** Mariana Castaneda-Guzman, Gabriel Mantilla-Saltos, Kris A. Murray, Robert Settlage, Luis E. Escobar

**Affiliations:** 1 Department of Fish and Wildlife Conservation, Virginia Polytechnic Institute and State University, Blacksburg, Virginia 24061 USA; 2 Escuela Superior Politécnica del Litoral, ESPOL, Facultad de Ciencias Naturales y Matemáticas, Guayaquil, Ecuador; 3 MRC Unit The Gambia at London School of Hygiene and Tropical Medicine, Fajara, The Gambia; 4 MRC Centre for Global Infectious Disease Analysis, School of Public Health, Imperial College London, London, UK; 5 Advanced Research Computing at Virginia Tech Carilion, Roanoke, Virginia USA; 6 Global Change Center, Virginia Tech, Blacksburg, VA USA; 7 Center for Emerging Zoonotic and Arthropod-borne Pathogens, Virginia Tech, Blacksburg, VA USA

**Keywords:** Environmental impact, Physical oceanography, Biogeography, Ecological modelling

## Abstract

Remote sensing satellite imagery has the potential to monitor and understand dynamic environmental phenomena by retrieving information about Earth’s surface. Marine ecosystems, however, have been studied with less intensity than terrestrial ecosystems due, in part, to data limitations. Data on sea surface temperature (SST) and Chlorophyll-*a* (Chlo-*a*) can provide quantitative information of environmental conditions in coastal regions at a high spatial and temporal resolutions. Using the exclusive economic zone of coastal regions as the study area, we compiled monthly and annual statistics of SST and Chlo-*a* globally for 2003 to 2020. This ready-to-use dataset aims to reduce the computational time and costs for local-, regional-, continental-, and global-level studies of coastal areas. Data may be of interest to researchers in the areas of ecology, oceanography, biogeography, fisheries, and global change. Target applications of the database include environmental monitoring of biodiversity and marine microorganisms, and environmental anomalies.

## Abstract (summary)

**Table:** 

Measurement(s)	temperature of sea surface • chlorophyll a
Technology Type(s)	satellite imaging
Factor Type(s)	geographic location • temporal interval
Sample Characteristic - Environment	sea coast
Sample Characteristic - Location	global

Machine-accessible metadata file describing the reported data: 10.6084/m9.figshare.16638235

## Background & Summary

Remote sensing, referring to the acquisition of information about the Earth’s surface through satellite imagery, has become a powerful tool for monitoring the environment and predicting risks associated with environmental changes^1–4^. From a plethora of applications, remotely sensed data have been used to detect landscape change^5–8^, assess biodiversity^9^, monitor carbon emissions^10,11^, predict infectious diseases^12–14^, and track marine coasts^15^. Marine ecosystems, however, have been studied with less intensity than terrestrial ecosystems due, in part, to data limitations. A limitation in the use of global-level remotely sensed data is how time-consuming it proves to be, given that sometimes complex data compilation, curation, standardization, and storage may require high-performance computational facilities^16,17^. An open-access, free-of-cost database of global ocean conditions is instrumental in advancing our understanding of coastal phenomena^12,15^.

A significant benefit of satellite-derived information is the historical archives of data^2,10,12^. Technological advances and innovative design have resulted in new generations of satellite sensors that monitor marine environments, such as the Moderate Resolution Imaging Spectroradiometer (MODIS). MODIS sensors are part of the National Aeronautics and Space Administration’s Earth Observing System onboard the Terra and Aqua satellites and were designed to provide measurements of global dynamics of terrestrial, freshwater, and marine ecosystems^18–20^. MODIS provides the longest standing observational marine time series data, given that both the Aqua and Terra satellites have been in orbit since the early 2000s, and it provides a larger set of marine variables for potential evaluation at the same spatial and temporal scale^18^. Nevertheless, there are other enhanced satellite instruments^21^, such as the Along Track Scanning Radiometer^22^, Suomi National Polar-orbiting Partnership^23^, Visible Infrared Imaging Radiometer Suite^24,25^, and Sentinel^26^, which offer opportunities for future multi-sensor marine variables.

Out of the possible marine variables derived from observations of MODIS, sea surface temperature (SST) and Chlorophyll-*a* (Chlo-*a*) have the potential to increase our understanding of abiotic (e.g., temperature) and biotic (e.g., primary productivity) ocean conditions^4,20^. SST measured by MODIS infrared radiometers is also referred to as the skin temperature of the ocean. This is because the radiance measured by infrared radiometers originates in the surface thermal skin layer of the ocean and not the water below as measured by *in situ* thermometers^27^. SST provides fundamental information on the global climate systems, and it is an essential parameter in weather prediction^28^. Chlo-*a* is a proxy for understanding fluctuations in algae and pigmented bacteria as it can elucidate photosynthetic activity in coastal systems^4,20,29^. The near-surface concentration of Chlo-*a* is calculated using an empirical relationship derived from *in situ* measurements, and the implementation of the standard O’Reilly band ratio OCx (e.g., OC3M, for the MODIS sensor) algorithm merged with the color index algorithm of Hu *et al*.^30,31^. SST and Chlo-*a* have been crucial in studies to reconstruct environmental phenomena, such as *Vibrio cholerae* emergence^13,32,33^, algae blooms^29,34,35^, El Niño and La Niña dynamics^36^, and coral bleaching^37^.

Satellite-derive data have many limitations given their sensitivity to absorption of solar isolation, heat exchange with the atmosphere, and sub-surface turbulence. Nevertheless, since these conditions are known and common, validation and uncertainty are estimated relative to *in situ* buoys to correct final datasets^38–40^. Satellite-derived data provide an opportunity to analyze large study areas during extended periods, at the cost of limiting the information to surface level. Complementary approaches may include the addition of more oceanic and atmospheric observations like bathymetry, wind direction, and wind speed^1^. We compiled remotely sensed data of monthly SST and Chlo-*a* from the exclusive economic zone (EEZ) of coastal areas globally for a 18-year period (2003–2020). Data were used to generate summary statistics at yearly and monthly composites. Code is included to update the database as data are released. This database can be downloaded freely through Figshare^41^.

## Methods

**Fig. 1:**
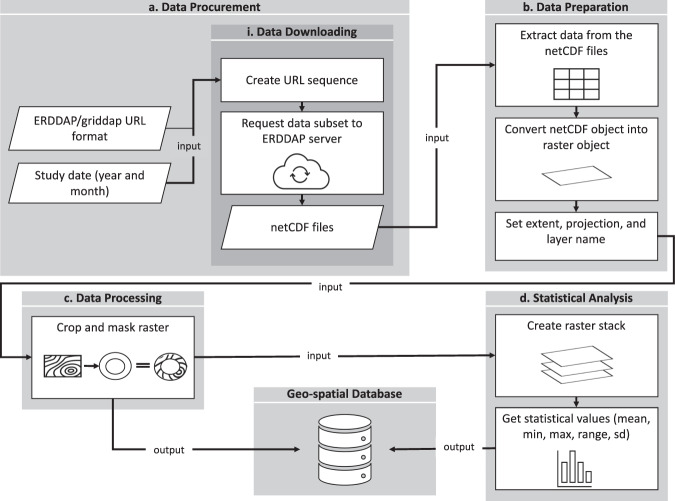
Workflow diagram. (**a**) Remotely-sensed data were downloaded from the NASA ERDDAP server in the form of NetCDF files. (**b**) Data were then transformed into a raster object. (**c**) Data were then cropped and masked to the exclusive economic zone and imported as GeoTIFF. (**d**) Data were analyzed to include statistical analyses and exported as raster files.

This section describes the procedures used to generate the individual data records that comprise the SST and Chlo-*a* databases. Data retrieval and analysis performed during the development of the database were executed using the statistical software R^42^. The SST and Chlo-*a* databases were developed in four stages: (a) data procurement, (b) preparation, (c) processing, and (d) analysis. The first two stages were associated with input data, while the third stage was applied specific methods to construct the core of each database. The fourth stage included the statistical analyses of the data. The methodological stages are summarized in Fig. 1 and described in detail below.

### Data procurement

The database is based on satellite observations derived from the MODIS satellite. The Terra and Aqua satellites have been orbiting around the Earth since their launch in 1999 and 2002, respectively, obtaining data of Earth’s surface every one to two days at three spatial resolutions (250, 500, 1000 m) and 36 spectral bands (from 0.405 to 14.385 µm). From the available atmospheric and oceanic observations made available from NASA’s Aqua Spacecraft, Sea Surface Temperature (SST) in °C and Chlorophyll-*a* (Chlo-*a*) in mg*m^−3^ were selected since they summarize major physical and biological phenomena. SST and Chlo-*a* are available at a temporal resolution of 1-day, 8-day, and monthly composites and a spatial resolution of ~4 km (Table 1).

**Table 1:** Data specifications for MODIS remotely-sensed data.

Database Title	Originator	Access	Dataset ID	Temporal range	Temporal resolution	Spatial resolution	Type	Format
SST, AQUA_MODIS, L3m.MO.SST.sst.4 km, Masked, SMI, NASA GSFC OBPG, R2019.0, Global, 0.04166°,	NASA Earth Observing System	https://coastwatch.pfeg.noaa.gov/erddap/griddap/erdMH1sstdmdayR20190SQ.html	erdMH1sstdmdayR20190SQ	2003-2020	Monthly Composite	4 km	Remotely-sensed	NetCDF
Chlorophyll-*a*, Aqua MODIS, NPP, L3SMI, Global	NASA Earth Observing System	https://coastwatch.pfeg.noaa.gov/erddap/griddap/erdMH1chlamday.html	erdMH1chlamday	2003-2020	Monthly Composite	4 km	Remotely-sensed	NetCDF

Original satellite-based imagery was collected by the MODIS instrument, part of the NASA Earth Observing System, and downloaded through the NASA’s ERDP server at a temporal resolution of monthly composite, from 2003 to 2020 and at a 4 km spatial resolution as NetCDF files.

SST and Chlo-*a*, among other environmental variables, can be accessed through National Oceanic and Atmospheric Administration’s (NOAA) Coastal Watch Environmental Research Division (ERD) Environmental Research Division Data Access Protocol (ERDDAP) data server, also known as the NOAA’s Coastal Watch. NOAA’s Coastal Watch is a program that provides timely access to near-real-time satellite data to monitor, restore, and manage coastal ocean resources, and the ERDDAP Data Server supports manual downloads through a web application and remote downloads from any computer program (e.g., MATLAB, R, JSONP, Python) of both gridded and tabular data^43^.

#### Data downloading

The remote request to the ERDDAP Data Server relies on the creation of specially formed URLs to query the server for a specific database. A URL consists of a root, a target, and a constraint expression^43^. To procure the inputs needed to assemble this database especially formed URLs were created through a programming algorithm in R (Auxiliary Materials^44^).

**The root** or base URLs that provided the location of the gridded database were obtained from the ERDDAP griddap documentation webpage (https://coastwatch.pfeg.noaa.gov/erddap/griddap/documentation.html) and remained constant in all requests for a specific database.

**The target** is the equivalent to the unique identifier or data set ID previously assigned by the ERDDAP (https://coastwatch.pfeg.noaa.gov/erddap/griddap), in conjunction with a specific data file type extension, for this study *.nc* was selected producing NetCDF-3 binary files with COARDS/CF/ACDD metadata. NetCDF, Network Common Data Form, files are recommended when using software tools to analyze geospatial data as they provide multidimensional scientific data in a standardized manner (https://coastwatch.pfeg.noaa.gov/erddap/griddap/documentation.html)^45,46^.

**The constraint expression** (or query) helped define the parameters, which correspond to the study period and spatial coverage. Regarding the first parameter, the study period comprised all available observations from the MODIS instrument aboard the Aqua satellite (i.e., monthly composites from 2003 to 2020). The spatial coverage was defined by the minimum and maximum latitude (i.e., 89.98°S to 89.98°N) and longitude (i.e., 179.98°W to 179.98°E) from the original satellite image for global coverage.

### Data preparation

Data within the NetCDF files were imported into R using the *RNetCDF* package^47^. A NetCDF object contains a list of at least four attributes: time, longitude, latitude, and the values of the variable being measured (i.e., SST and Chlo-*a*). The attribute corresponding to the specific variable being measured was extracted from the NetCDF object and transformed into a raster object using the *RNetCDF* and *raster* packages in R^48^. A raster object consists of a matrix of cells (i.e., pixels) organized into rows and columns where each cell contains a value representing information (i.e., temperature and pigmentation) and the metadata corresponding to spatial information of object^49^.

As the last piece of the data preparation process, the extent of the raster was verified to match that of the original satellite data. Extent was set to latitude and longitude of 89.98°S to 89.98°N and 179.98°W to 179.98°E, respectively. The coordinate reference system (CRS) was defined to be relative to the WGS84 datum for easy manipulation by the end user.

### Data processing

A significant feature of the SST and Chlo-*a* databases is the addition of the segmentation by the world’s exclusive economic zone (EEZ). EEZ is a marine zone within 200 nautical miles from a country’s coastline where each country claims jurisdiction for economic activities^50^. Given the oceanographic nature of the data, focusing on the 200-mile buffer of EEZ provides a more comprehensive explanation of oceanic changes, with the potential to promote the development of ocean planning initiatives directly influencing human settlements on the coasts. To represent the EEZ, a geospatial vector file in shapefile format was constructed by delimiting a buffer of ~200 miles off coastlines globally.

**Fig. 2:**
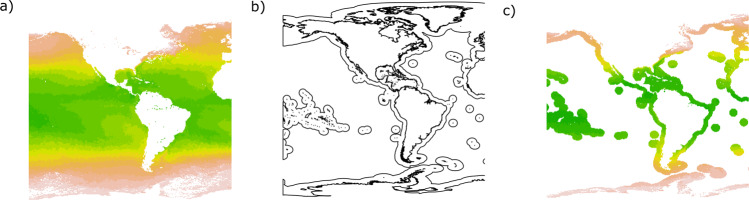
Data masking and cropping. Example of masking and cropping a raster. (**a**) Raster from original NetCDF. (**b**) Economic Exclusive Zone (solid lines). (**c**) Raster after crop and mask.

The EEZ regions were defined using the functions *crop* and *mask* from the *raster*^48^ package. The function *mask* allowed to place the area of interest (i.e., the EEZ) on top of each monthly raster, assigning no value to cells outside of the area of interest, while the function *crop* ensured that each raster matched the extent of that of the area of interest (Fig. 2). The core database included 408 individual rasters cropped and masked to the EEZ of each country.

### Statistical analysis

Complementary to the core database, data were treated as an *m* by *n* matrix, where *m* represents the years and *n* represents the months and stacked in two distinct ways (1) in yearly composites and (2) monthly composites.

1 $${\sum }_{j=1}^{n}\,{x}_{ij}\;{\rm{for}}\;i=1,\ldots ,\;m$$

2 $${\sum }_{i=1}^{m}\,{x}_{ij}\;{\rm{for}}\;j=1,\ldots ,\;n$$

We created the annual and monthly stacks by using *stack* function in the *raster* package^48^. The mean, range, maximum, minimum, and standard deviation values were estimated for annual and monthly SST and Chlo-*a*. We obtained a total of 90 rasters for the yearly composites (18 years, five different statistics) and 60 rasters for the monthly summaries (12 months, five different statistics).

## Data Records

Final data are provided in the form of GeoTIFFs for the EEZs boundaries and statistical analysis results^41^. Data can be downloaded based on annually, monthly, or as summary composites of the 18-years period. Data can also be updated using the code included in the Auxiliary Material in Figshare^44^.

## Technical Validation

Remotely sensed environmental observations from the MODIS instrument, including SST and Chlo-*a*, have been validated profusely by the scientific community against a number of models and *in situ* measurements^51–58^ and used in a diverse set of studies^13,14,19,59–67^. For instance, validation of the SST observation uses accurate ship-based infrared radiometers and differing and moored buoys with thermometers a meter of depth^38,56,57^. NASA’s standard processing and distribution of the SST products are performed using software developed by the Ocean Biology Processing Group^18^. SST products are validated internally by NASA using a collocated matchup database of *in situ* observations that are collected within 30 minutes of an overpass and 10 km of a pixel. MODIS SST observations represent the thermal skin layer of the ocean, which is <1 mm thick and is cooler than the underlying water due to vertical heat flux^68,69^. At night or when wind speeds are greater than ~6 m/s, the relationship between the skin temperature and the subsurface are nearly equal. It is under these conditions that validation and uncertainty estimates relative to sub-surface *in situ* buoys are typically reported^20,38^. The estimation vs. observation relationship, however, can be very variable under conditions of low wind speeds and reduced sub-surface turbulence^21,70^. Furthermore, NASA MODIS uses a collection of cloud classification algorithms to indicate when a pixel corresponds to clear sky conditions (i.e., no cloud coverage). The most recent cloud-classification method is the Alternating Decision Tress^71^. Other SST observations validations tests include a regional ice test, where reflectance thresholds are determined using the Sentinel-2 MSI calibrated reflectance^72^ and correction of dust contamination^73^.

MODIS Chlo-*a* observations are derived from the O’Reilly OC3M algorithm and the Hu color index^30,31^. The algorithm is calculated using an empirical relationship from *in situ* measurements and remote sensing reflectance in the blue-to-green region of the visible spectrum. Level 3 MODIS data may provide biased minima and maxima values during errors in the observation that, for example, has some cloud contamination or sunlight affecting the value captured by the sensor. Due to potential atmospheric contamination some regions could have a limited number of observations from which to estimate the monthly values, which increases uncertainty. There is an estimated ± 35% nominal uncertainty related to the OC3M algorithm used to derive the global Chlo-*a* product. Nevertheless, error could increase in optically complex waters like those present in coastal areas^74,75^.

We performed a data validation procedure comparing MODIS observation of SST and Chlo-*a* against gold-standard sensors. More specifically, we compared MODIS data against SST data from Sentinel-3^76^ during the year 2020. We found that data from MODIS and Sentinel-3 were statistically indistinguishable with a Pearson correlation coefficient of *r* = 0.99 for the annual mean, minimum, and maximum composites (*R*^2^ = 0.99, *p* < 0.05; Supplementary Fig. S1). Additionally, Chlo-*a* data were evaluated by comparing MODIS data against SeaWiFS^30^ observations for the year 2010, when the SeaWiFS satellite ended operations. We found that MODIS Chlo-*a* data were significantly correlated with SeaWiFS Chlo-*a* data but with less strength than for SST evaluations. More specifically, correlation was *r* = 0.83 (*R*^2^ = 0.67, *p* < 0.05) for the mean, *r* = 0.71 (*R*^2^ = 0.53, *p* < 0.05) for the maximum, and *r* = 0.76 (*R*^2^ = 0.52, *p* < 0.05) for the minimum Chlo-*a* composites (Fig. S2). Together, these results suggest that MODIS data have a robust representation of environmental conditions in global coastal waters, at least when compared against gold-standard datasets of SST and Chlo-*a*.

## Usage Notes

**Fig. 3:**
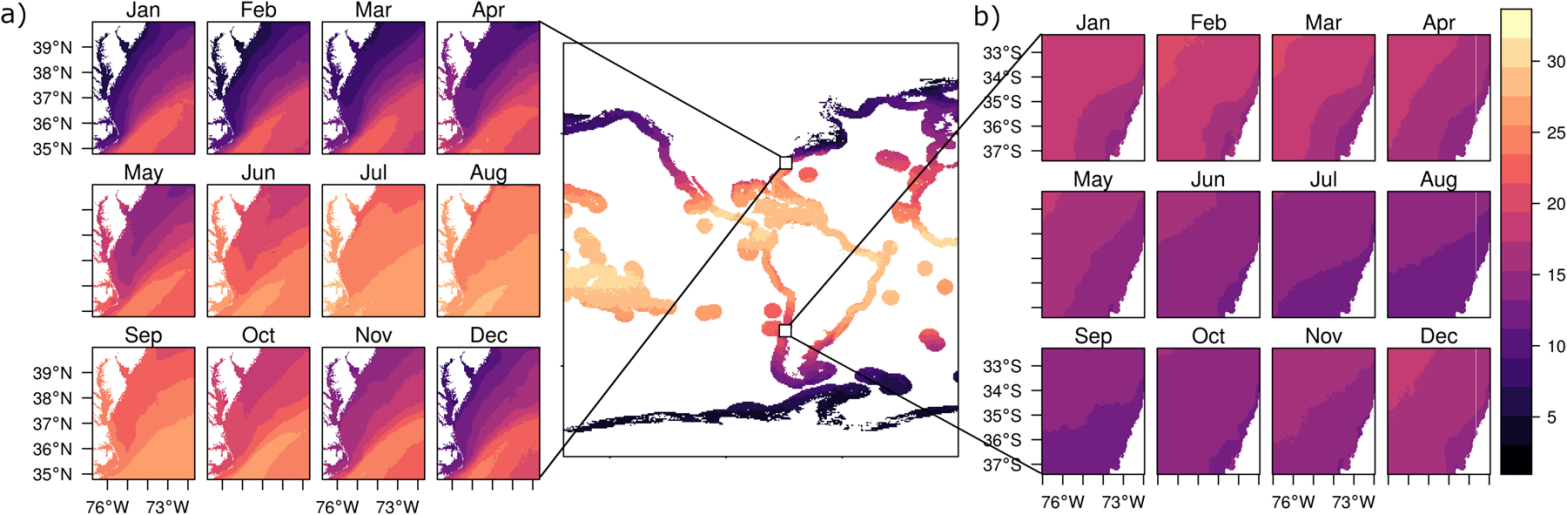
Sea surface temperature mean monthly values from 2003–2020. (**a**) Temperate zone monthly averages between the years 2003–2020 (east coast of the United States). (**b**) Subtropical zone monthly averages between the years 2003–2020 (coast of Chile).

**Fig. 4:**
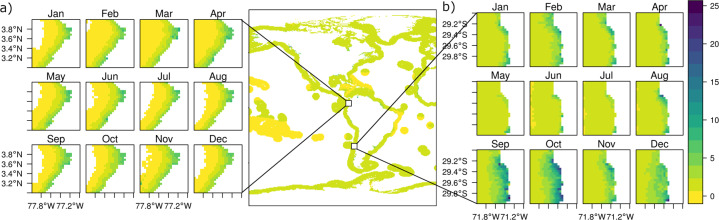
Chlorophyll-*a* mean monthly values from 2003–2020. (**a**) Tropical zone monthly averages between the years 2003–2020 (coast of Ecuador and Colombia). (**b**) Subtropical zone monthly averages between the years 2003–2020 (coast of Chile).

The proposed use of this dataset is for coarse-scale, regional or global-level studies of coastal environmental conditions. Fine-scale assessments of SST and Chlo-*a* are warranted to improve accuracy and detail of these variables for local-level applications. The data can be used to identify anomalies for SST and Chlo-*a* at local, regional, and global levels. The example demonstrates SST and Chlo-*a* data explorations in tropical and temperate localities, identifying patterns along time (Fig. 3). Areas in the mid-Atlantic region of the United States show an increase in mean SST during the month of June to October (Fig. 3a), while areas in the subtropics of the Americas (i.e., Ecuador and Colombia) reveal cooler temperatures during the same period (Fig. 3b). Additional exploration of the data in tropical and subtropical zones of different latitude reveal that Chlo-*a* increases from September to December (Fig. 4b). Contrarily, in the tropics, Chlo-*a* concentration increases between March and May (Fig. 4a).

## Supplementary information

Supplementary Material

## Data Availability

Code in R language to recreate the database and the figures in the Usage Notes is available on Figshare^41^.

## References

[1] Horning, N., Robinson, J. A., Sterling, E. J., Turner, W. & Spector, S. *Remote sensing for ecology and conservation*. *Techniques in Ecology & Conservation Series* (Oxford University Press, 2010).

[2] Li J (2020). A review of remote sensing for environmental monitoring in China. Remote Sens..

[3] Carter, W. D. & Paulson, R. W. *Introduction to monitoring dynamic environmental phenomena of the world using satellite data collection systems*. (U.S. Geological Survey, 1979).

[4] Nurdin S, Mustapha MA, Lihan T (2013). The relationship between sea surface temperature and chlorophyll-*a* concentration in fisheries aggregation area in the archipelagic waters of spermonde using satellite images. AIP Conf. Proc..

[5] Ward D, Phinn SR, Murray AT (2000). Monitoring growth in rapidly urbanizing areas using remotely sensed data. Prof. Geogr..

[6] Singh A (1989). Review article: Digital change detection techniques using remotely-sensed data. Int. J. Remote Sens..

[7] Dewan AM, Yamaguchi Y (2009). Land use and land cover change in Greater Dhaka, Bangladesh: Using remote sensing to promote sustainable urbanization. Appl. Geogr..

[8] Green K, Kempka D, Lackey L (1994). Using remote sensing to detect and monitor land-cover and land-use change. Photogramm. Eng. Remote Sens..

[9] Nagendra H (2001). Using remote sensing to assess biodiversity. Int. J. Remote Sens..

[10] Rosenqvist Å, Milne A, Lucas R, Imhoff M, Dobson C (2003). A review of remote sensing technology in support of the Kyoto Protocol. Environ. Sci. Policy.

[11] Liu J (1997). A process-based boreal ecosystem productivity simulator using remote sensing inputs. Remote Sens. Environ..

[12] Colwell RR (1996). Global climate and infectious disease: The cholera paradigm. Science.

[13] Escobar LE (2015). A global map of suitability for coastal *Vibrio cholerae* under current and future climate conditions. Acta Trop..

[14] Watts N (2019). The 2019 report of The Lancet Countdown on health and climate change: Ensuring that the health of a child born today is not defined by a changing climate. Lancet.

[15] Alesheikh AA, Ghorbanali A, Nouri N (2007). Coastline change detection using remote sensing. Int. J. Environ. Sci. Technol..

[16] Specter C, Gayle D (1990). Managing technology transfer for coastal zone development: Caribbean experts identify major issues. Int. J. Remote Sens..

[17] Green EP, Mumby PJ, Edwards AJ, Clark CD (1996). A review of remote sensing for the assessment and management of tropical coastal resources. Coast. Manag..

[18] NASA. MODIS (Moderate Resolution Imaging Spectroradiometer). https://modis.gsfc.nasa.gov/about/ (2021).

[19] Kilpatrick KA (2015). A decade of sea surface temperature from MODIS. Remote Sens. Environ..

[20] Esaias WE (1998). An overview of MODIS capabilities for ocean science observations. IEEE Trans. Geosci. Remote Sens..

[21] Donlon CJ (2002). Toward improved validation of satellite SST measurements for climate research. J. Clim..

[22] Minnett, P. J. Satellite infrared scanning radiometers — AVHRR and ATSR/M. in *Microwave Remote Sensing for Oceanographic and Marine Weather-Forecast Models* 141–163 (Springer Netherlands, 1990).

[23] Hillger D (2013). First-Light Imagery from Suomi NPP VIIRS. Bull. Am. Meteorol. Soc..

[24] O’Brien, J. From MODIS to VIIRS - Making the Switch for Air Quality Professionals. *NASA Earth Science/Applied Science* https://appliedsciences.nasa.gov/our-impact/news/modis-viirs-making-switch-air-quality-professionals (2020).

[25] Minnett, P. J., Evans, R. H., Podestá, G. P. & Kilpatrick, K. A. Sea-surface temperature from Suomi-NPP VIIRS: Algorithm development and uncertainty estimation. in *SPIE 9111*, *Ocean Sensing and Monitoring VI* (eds. Hou, W. W. & Arnone, R. A.) 91110C (2014).

[26] Drusch M (2012). Sentinel-2: ESA’s Optical High-Resolution Mission for GMES Operational Services. Remote Sens. Environ..

[27] Donlon C (2007). The global ocean data assimilation experiment high-resolution sea surface temperature pilot project. Bull. Am. Meteorol. Soc..

[28] NOAA. Ocean Facts: Why do scientists measure sea surface temperature? https://oceanservice.noaa.gov/facts/sea-surface-temperature.html (2020).

[29] Wei GF, Tang DL, Wang S (2008). Distribution of chlorophyll and harmful algal blooms (HABs): A review on space based studies in the coastal environments of Chinese marginal seas. Adv. Sp. Res..

[30] O’Reilly JE (1998). Ocean color chlorophyll algorithms for SeaWiFS. J. Geophys. Res. Ocean..

[31] Hu C, Lee Z, Franz B (2012). Chlorophyll a algorithms for oligotrophic oceans: A novel approach based on three-band reflectance difference. J. Geophys. Res. Ocean..

[32] Vezzulli L (2016). Climate influence on Vibrio and associated human diseases during the past half-century in the coastal North Atlantic. Proc. Natl. Acad. Sci..

[33] Lipp EK, Huq A, Colwell RR (2002). Effects of global climate on infectious disease: The Cholera model. Clin. Microbiol. Rev..

[34] Grimes JD (2014). Viewing marine bacteria, their activity and response to environmental drivers from orbit: Satellite remote sensing of bacteria. Microb. Ecol..

[35] Shen, L., Xu, H. & Guo, X. Satellite remote sensing of harmful algal blooms (HABs) and a potential synthesized framework. *Sensors* **12**, 7778–803 (2012). 10.3390/s120607778 PMC3436001 22969372

[36] Hayashi M, Jin F, Stuecker MF (2020). Dynamics for El Niño-La Niña asymmetry constrain equatorial-Pacific warming pattern. Nat. Commun..

[37] Hughes, T. P. *et al*. Spatial and temporal patterns of mass bleaching of corals in the Anthropocene. *Science* **359**, 80–83 (2018). 10.1126/science.aan8048 29302011

[38] Minnett, P. J. *et al*. Sea-surface temperature measurements from the moderate-resolution imaging spectroradiometer (MODIS) on Aqua and Terra. in *IEEE International Geoscience and Remote Sensing Symposium Proceedings. 2004* **7**, 4576–4579 (2004).

[39] Minnett, P. J. The validation of sea surface temperature retrievals from spaceborne infrared radiometers. in *Oceanography from Space* (Springer Netherlands, 2010).

[40] Minnett PJ, Corlett GK (2012). A pathway to generating climate data records of sea-surface temperature from satellite measurements. Deep Sea Res. Part II Top. Stud. Oceanogr..

[41] Castaneda-Guzman M, Mantilla-Saltos G, Murray KA, Settlage R, Escobar LE (2021). Figshare.

[42] R Core Team. R: A Language and Environment for Statistical Computing. (2020).

[43] NOAA. National Oceanic and Atmospheric Administration (NOAA) Coastal Watch. https://coastwatch.pfeg.noaa.gov/erddapinfo/ (2021).

[44] Castaneda-Guzman M, Mantilla-Saltos G, Murray KA, Settlage R, Escobar LE (2021). Figshare.

[45] Stanford. Best practices for file formats. https://library.stanford.edu/research/data-management-services/data-best-practices/best-practices-file-formats (2021).

[46] UCAR Community Programs. Network Common Data Form (NetCDF). https://www.unidata.ucar.edu/software/netcdf/ (2021).

[47] Michna, P. & Woods, M. RNetCDF: Interface to ‘NetCDF’ Datasets. (2019).

[48] Hijmans, R. J. raster: Geographic Data Analysis and Modeling. (2020).

[49] ArcGIS. What is a raster data? https://desktop.arcgis.com/en/arcmap/10.3/manage-data/raster-and-images/what-is-raster-data.htm (2021).

[50] United Nations. *United Nations Convention on the Law of the Sea*. 1833 U.N.T.S. **397** (1982).

[51] Tilstone GH (2013). Assessment of MODIS-Aqua chlorophyll-*a* algorithms in coastal and shelf waters of the eastern Arabian Sea. Cont. Shelf Res..

[52] Hoge FE (2003). Validation of Terra-MODIS phytoplankton chlorophyll fluorescence line height. I. Initial airborne Lidar results. Appl. Opt..

[53] Remer LA (2002). Validation of MODIS aerosol retrieval over ocean. Geophys. Res. Lett..

[54] Gentemann CL (2014). Three way validation of MODIS and AMSR-E sea surface temperatures. J. Geophys. Res. Ocean..

[55] Fang H, Wei S, Liang S (2012). Validation of MODIS and CYCLOPES LAI products using global field measurement data. Remote Sens. Environ..

[56] Hosoda K, Murakami H, Sakaida F, Kawamura H (2007). Algorithm and validation of sea surface temperature observation using MODIS sensors aboard terra and aqua in the western North Pacific. J. Oceanogr..

[57] Hao Y (2017). Validation of MODIS sea surface temperature product in the coastal waters of the Yellow Sea. IEEE J. Sel. Top. Appl. Earth Obs. Remote Sens..

[58] Sims, D. A. *et al*. On the use of MODIS EVI to assess gross primary productivity of North American ecosystems. *J. Geophys. Res. Biogeosciences* **111** (2006).

[59] Miles TN, He R (2010). Temporal and spatial variability of Chl-a and SST on the South Atlantic Bight: Revisiting with cloud-free reconstructions of MODIS satellite imagery. Cont. Shelf Res..

[60] Ma, S., Zhang, X., Ding, C., Han, W. & Lu, Y. Comparison of the spatiotemporal variation of Chl-a in the East China Sea and Bohai Sea based on long time series satellite data. in *2021 9th International Conference on Agro-Geoinformatics (Agro-Geoinformatics)* 1–6 (2021).

[61] Watts, N. *et al*. The 2020 report of The Lancet Countdown on health and climate change: Responding to converging crises. *Lancet* **6736** (2020). 10.1016/S0140-6736(20)32290-X PMC7616803 33278353

[62] Moradi M, Kabiri K (2015). Spatio-temporal variability of SST and Chlorophyll-*a* from MODIS data in the Persian Gulf. Mar. Pollut. Bull..

[63] Golder MR (2021). Chlorophyll-*a*, SST and particulate organic carbon in response to the cyclone Amphan in the Bay of Bengal. J. Earth Syst. Sci..

[64] Minnett, P. J., Evans, R. H., Kearns, E. J. & Brown, O. B. Sea-surface temperature measured by the Moderate Resolution Imaging Spectroradiometer (MODIS). in *IEEE International Geoscience and Remote Sensing Symposium* vol. 2, 1177–1179 (IEEE, 2002).

[65] Qin H, Chen G, Wang W, Wang D, Zeng L (2014). Validation and application of MODIS-derived SST in the South China Sea. Int. J. Remote Sens..

[66] Saulquin B, Gohin F, Garrello R (2011). Regional Objective Analysis for Merging High-Resolution MERIS, MODIS/Aqua, and SeaWiFS Chlorophyll-a Data From 1998 to 2008 on the European Atlantic Shelf. IEEE Trans. Geosci. Remote Sens..

[67] Chen J, Quan W (2013). An improved algorithm for retrieving chlorophyll-*a* from the Yellow River Estuary using MODIS imagery. Environ. Monit. Assess..

[68] Hanafin, J. A. & Minnett, P. J. Thermal profiling of the sea surface skin layer using FTIR measurements. in *Gas Transfer at Water Surfaces* 161–166 (Blackwell Publishing, 2002).

[69] Wong EW, Minnett PJ (2018). The response of the ocean thermal skin layer to variations in incident infrared radiation. J. Geophys. Res. Ocean..

[70] Ward B (2006). Near-surface ocean temperature. J. Geophys. Res..

[71] Kilpatrick KA, Podestá GP, Evans R (2001). Overview of the NOAA/NASA advanced very high resolution radiometer Pathfinder algorithm for sea surface temperature and associated matchup database. J. Geophys. Res. Ocean..

[72] Hollstein A, Segl K, Guanter L, Brell M, Enesco M (2016). eady-to-use methods for the detection of clouds, cirrus, snow, shadow, water and clear sky pixels in Sentinel-2 MSI images. Remote Sens..

[73] Luo B, Minnett PJ, Gentemann C, Szczodrak G (2019). Improving satellite retrieved night-time infrared sea surface temperatures in aerosol contaminated regions. Remote Sens. Environ..

[74] Moore TS, Campbell JW, Dowell MD (2009). A class-based approach to characterizing and mapping the uncertainty of the MODIS ocean chlorophyll product. Remote Sens. Environ..

[75] Pieri M (2015). Assessment of three algorithms for the operational estimation of [CHL] from MODIS data in the Western Mediterranean Sea. Eur. J. Remote Sens..

[76] Tilstone GH (2021). Performance of Ocean Colour Chlorophyll-a algorithms for Sentinel-3 OLCI, MODIS-Aqua and Suomi-VIIRS in open-ocean waters of the Atlantic. Remote Sens. Environ..

