# Supplementary material for: A database of global coastal conditions
Source: Sci Data. 2021 Nov 26;8:304. doi: 10.1038/s41597-021-01081-9 (PMC8626420; doi:10.1038/s41597-021-01081-9)
Supplement: Supplementary file 1 — Supplementary Material [file 41597_2021_1081_MOESM1_ESM.docx]

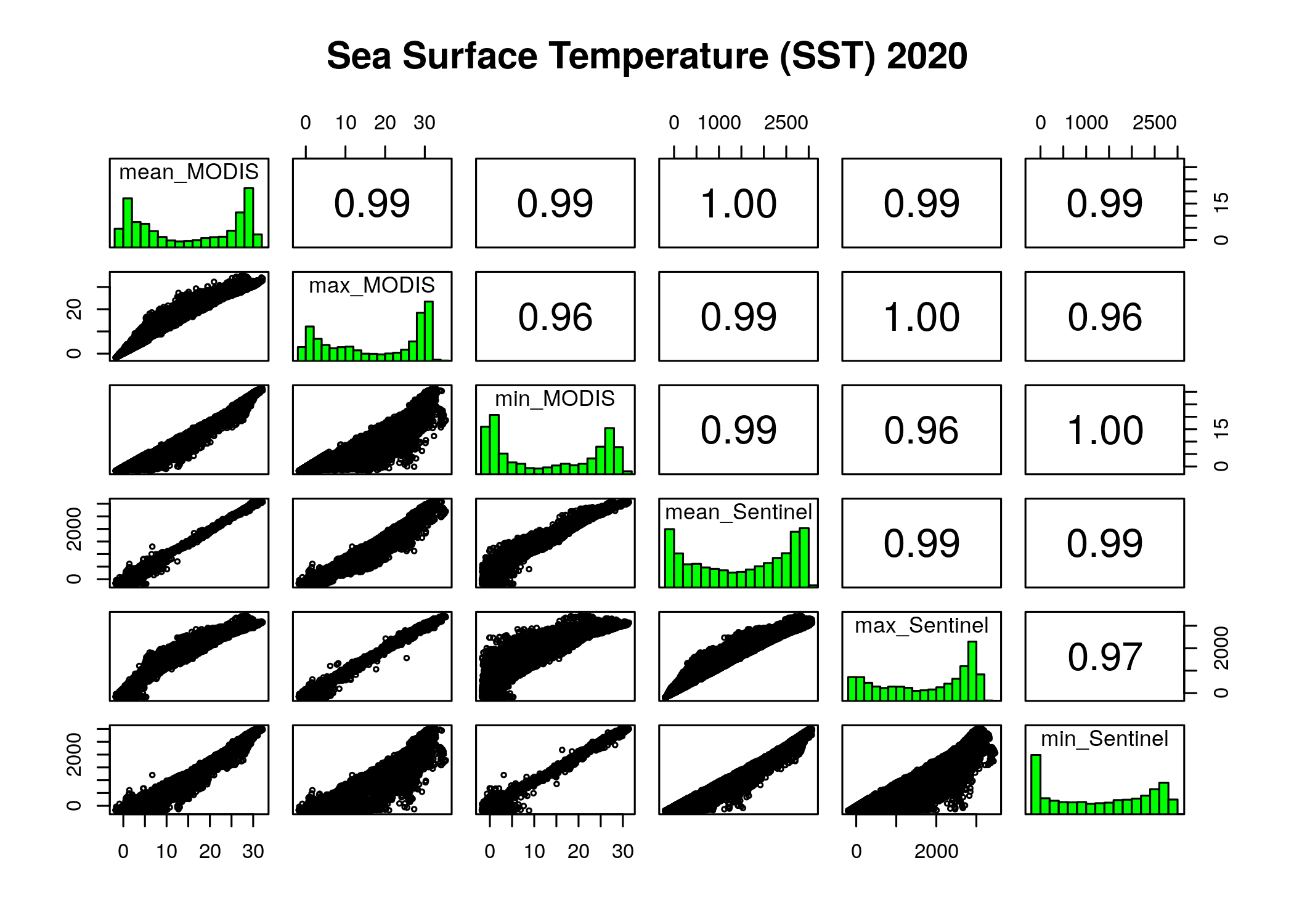


**Figure S1. Sea surface temperature correlation between MODIS and Sentinel-3 data during the year 2020.** Correlation for mean, minimum, and maximum, between both sensors results in a high positive correlation with a Pearson correlation coefficient of r < 0.99 for all scenarios.


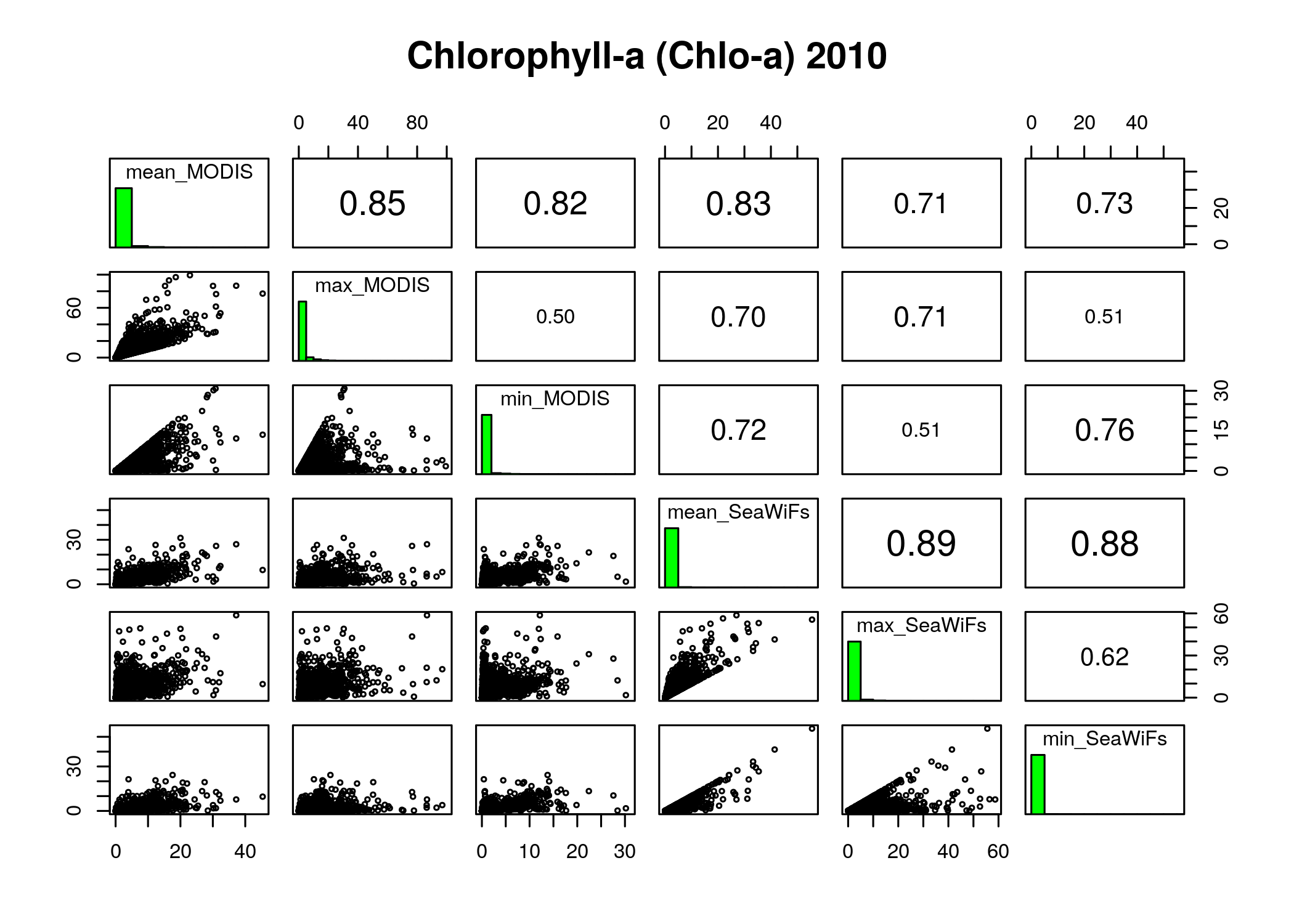


**Figure S2. Chlorophyll-*a* correlation between MODIS and SeaWiFS data during the year 2010.** Correlation for mean, minimum, and maximum, between both sensors results in a high positive correlation with a Pearson correlation coefficient of r <0.99 for all scenarios.

**Table S1. Linear regression and correlation model results between MODIS data against SST data from Sentinel-3 during the year 2020.** Linear regression and correlation results for mean, minimum, and maximum values from pixels of global datasets of MODIS and Sentinel-3 sensors (DF: degrees of freedom; RSE: residual standard error; r: Pearson correlation coefficient)

| Statistic | DF | R^2^ | p-value | RSE | r |
| --- | --- | --- | --- | --- | --- |
| Mean | 9832726 | 0.997 | <2e-16 | 0.591 | 0.999 |
| Min | 9832726 | 0.996 | <2e-16 | 0.771 | 0.998 |
| Max | 9832726 | 0.996 | <2e-16 | 0.717 | 0.998 |

**Table S1. Linear regression and correlation model results between MODIS data against CHLO-*a* data from SeaWiFS during the year 2010.** Linear regression and correlation results for mean, minimum, and maximum values from pixels of global datasets of MODIS and Sentinel-3 sensors (DF: degrees of freedom; RSE: residual standard error; r: Pearson correlation coefficient)

| Statistic | DF | R^2^ | p-value | RSE | r |
| --- | --- | --- | --- | --- | --- |
| Mean | 8943745 | 0.671 | <2e-16 | 1.135 | 0.819 |
| Min | 8943745 | 0.529 | <2e-16 | 0.802 | 0.727 |
| Max | 8943745 | 0.521 | <2e-16 | 3.019 | 0.722 |


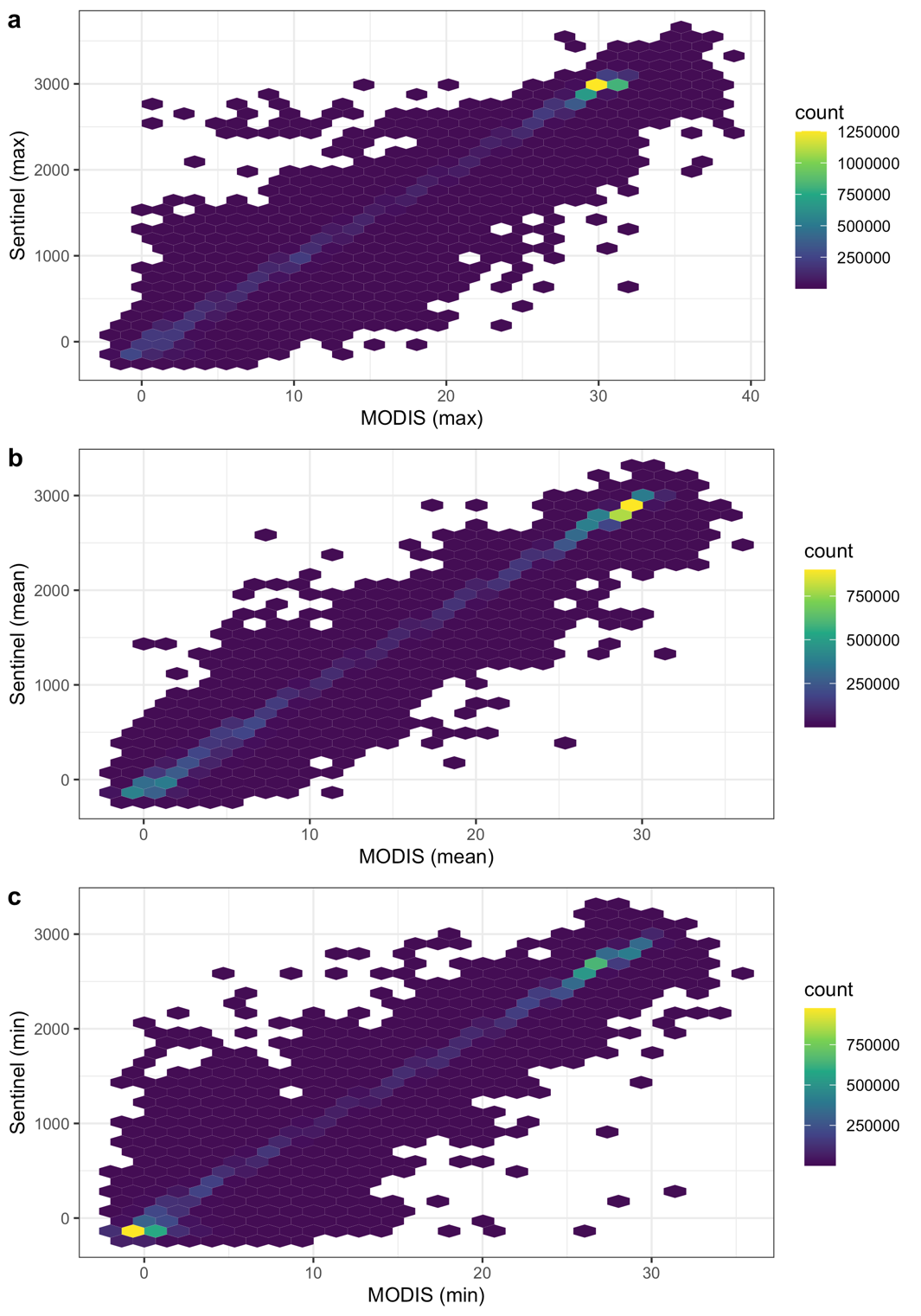


**Figure S3. Hexbin plot between MODIS and Sentinel-3 SST data during the year 2020.** The color of each hexagonal region denotes the number of pixels per value (i.e., density). Purple denotes a lower density of points, while green and yellow denote a higher density of points.


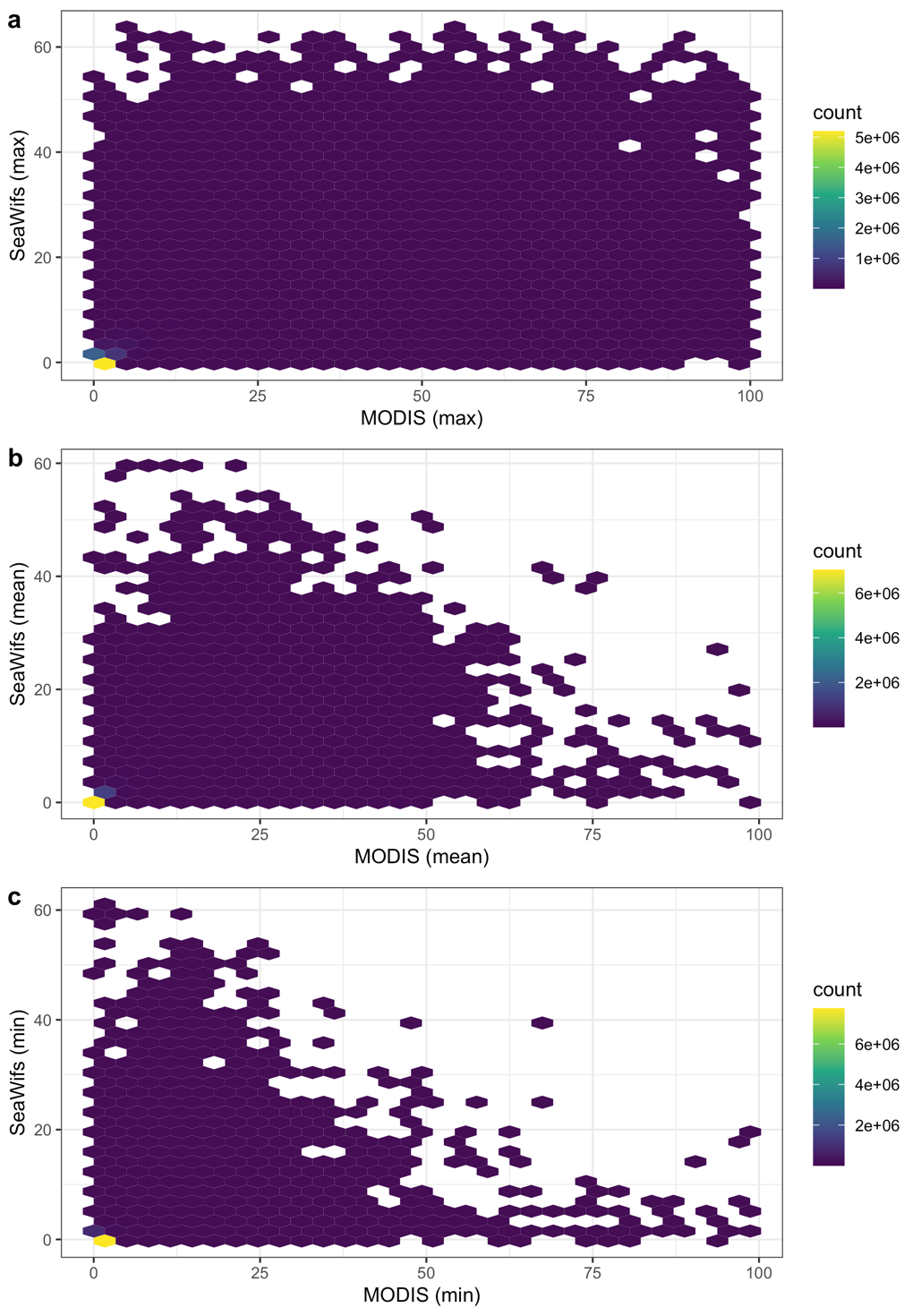


**Figure S4. Hexbin plot between MODIS and SeaWiFS data during the year 2010.** The color of each hexagonal region denotes the number of pixels per value (i.e., density). Purple denotes a lower density of points, while green and yellow denote a higher density of points.
